# Profiles of 5α-Reduced Androgens in Humans and Eels: 5α-Dihydrotestosterone and 11-Ketodihydrotestosterone Are Active Androgens Produced in Eel Gonads

**DOI:** 10.3389/fendo.2021.657360

**Published:** 2021-03-23

**Authors:** Takashi Yazawa, Hiroyuki Inaba, Yoshitaka Imamichi, Toshio Sekiguchi, Junsuke Uwada, Mohammad Sayful Islam, Makoto Orisaka, Daisuke Mikami, Takanori Ida, Takahiro Sato, Yoshimichi Miyashiro, Satoru Takahashi, Md. Rafiqul Islam Khan, Nobuo Suzuki, Akihiro Umezawa, Takeshi Kitano

**Affiliations:** ^1^ Department of Biochemistry, Asahikawa Medical University, Hokkaido, Japan; ^2^ Department of Biological Sciences, Graduate School of Science and Technology, Kumamoto University, Kumamoto, Japan; ^3^ Freshwater Resources Research Center, Aichi Fisheries Research Institute, Aichi, Japan; ^4^ Department of Pharmacology, Asahikawa Medical University, Hokkaido, Japan; ^5^ Noto Marine Laboratory, Institute of Nature and Environmental Technology, Division of Marine Environmental Studies, Kanazawa University, Ishikawa, Japan; ^6^ Department of Obstetrics-Gynecology, University of Fukui, Fukui, Japan; ^7^ Department of Nephrology, University of Fukui, Fukui, Japan; ^8^ Center for Animal Disease Control, University of Miyazaki, Miyazaki, Japan; ^9^ Molecular Genetics, Institute of Life Sciences, Kurume University, Fukuoka, Japan; ^10^ ASKA Pharma Medical Co., Ltd., Kanagawa, Japan; ^11^ Department of Pediatrics, Asahikawa Medical University, Hokkaido, Japan; ^12^ Department of Pharmacy, University of Rajshahi, Rajshahi, Bangladesh; ^13^ Department of Reproduction, National Research Institute for Child Health and Development, Tokyo, Japan

**Keywords:** DHT, 11KDHT, androgen receptor, 5α-reductase, testosterone

## Abstract

Although 11-ketotestosterone (11KT) and testosterone (T) are major androgens in both teleosts and humans, their 5α-reduced derivatives produced by steroid 5α-reductase (SRD5A/srd5a), i.e., 11-ketodihydrotestosterone (11KDHT) and 5α-dihydrotestosterone (DHT), remains poorly characterized, especially in teleosts. In this study, we compared the presence and production of DHT and 11KDHT in Japanese eels and humans. Plasma 11KT concentrations were similar in both male and female eels, whereas T levels were much higher in females. In accordance with the levels of their precursors, 11KDHT levels did not show sexual dimorphism, whereas DHT levels were much higher in females. It is noteworthy that plasma DHT levels in female eels were higher than those in men. In addition, plasma 11KDHT was undetectable in both sexes in humans, despite the presence of 11KT. Three srd5a genes (*srd5a1*, *srd5a2a* and *srd5a2b*) were cloned from eel gonads. All three *srd5a* genes were expressed in the ovary, whereas only both srd5a2 genes were expressed in the testis. Human *SRD5A1* was expressed in testis, ovary and adrenal, whereas *SRD5A2* was expressed only in testis. Human SRD5A1, SRD5A2 and both eel srd5a2 isoforms catalyzed the conversion of T and 11KT into DHT and 11KDHT, respectively, whereas only eel srd5a1 converted T into DHT. DHT and 11KDHT activated eel androgen receptor (ar)α-mediated transactivation as similar fashion to T and 11KT. In contrast, human AR and eel arβ were activated by DHT and11KDHT more strongly than T and 11KT. These results indicate that in teleosts, DHT and 11KDHT may be important 5α-reduced androgens produced in the gonads. In contrast, DHT is the only major 5α-reduced androgens in healthy humans.

## Introduction

**Figure 1:**
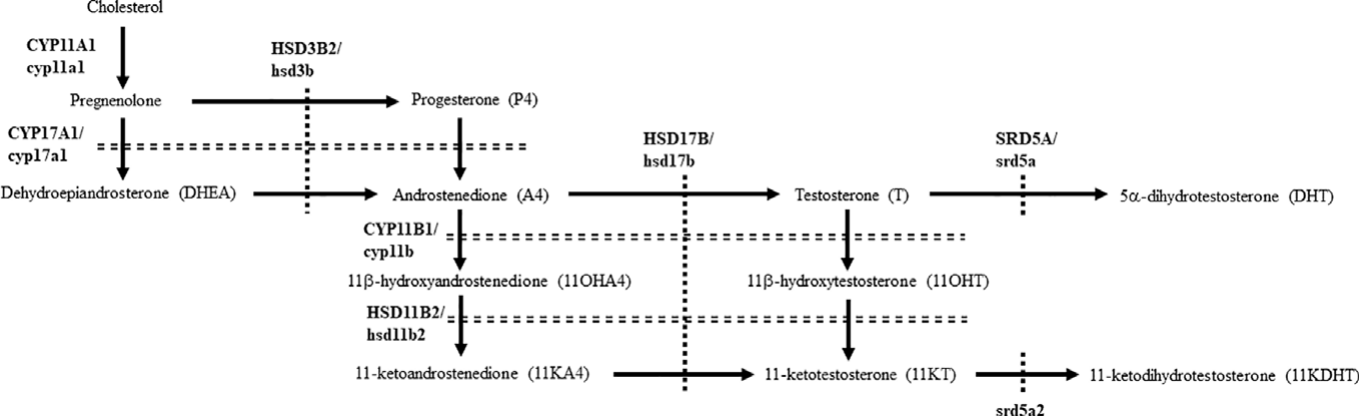
Pathways for producing 11-oxygenated androgens under physiological conditions in humans and teleosts. Human steroidogenic enzymes are indicated by capital letters, whereas teleost counterparts are indicated by small letters.

Androgens are sex steroid hormones that play a role in various physiological processes *via* pathways involving the androgen receptor (AR) (1). Testosterone (T) is the most important androgen in various animal species. It is produced from cholesterol in a series of steps by cytochrome P450 hydroxylases and hydroxysteroid dehydrogenases (HSDs) (**Figure 1**) (2, 3). Although T is able to strongly activate AR-mediated transactivation, it is converted into a more potent androgen, 5α-dihydrotestosterone (DHT), by steroid 5α-reductases (SRD5A) in steroidogenic tissues and peripheral tissues. Among the five human SRD5A genes, SRD5A1 and SRD5A2 play important roles in DHT production in gonads and peripheral tissues. SRD5A2 is strongly expressed in male tissues, including prostate and epididymis for producing DHT during development (4). Therefore, mutations of the *SRD5A2* gene cause 46, XY disorders of sex development, resulting from low DHT production (5–8). SRD5A1 mainly plays roles in the production of neurosteroids involved in anxiety and sexual behavior (4). It also has activity in conversion of T into DHT, although to a lesser extent than that of SRD5A2 (9). In fact, *Srd5a1* KO mice showed the abnormalities in these behaviors (10–12), and also partial feminization of the male skeleton (13).

In teleosts, 11-ketotestosterone (11KT), member of the 11-oxygenated class of androgens, is a major active androgen mainly produced in gonads (14, 15). It is involved in sex differentiation (16), spermatogenesis (17, 18), and oocyte growth (19–21). Although 11KT was regarded as a teleost-specific androgen, it is also a major androgens in some mammals, such as humans, non-human primates, pigs and guinea pigs (22–25). Although 11KT strongly activates both mammalian and teleostan AR/ar (22, 23, 26–29), it is able to be converted into the even more active form, 11-ketodihydrotestosterone (11KDHT), by SRD5A1 and SRD5A2 (30). 11KDHT is produced in prostate cancer cells (30, 31), although it is unclear whether it is also produced in healthy humans. Furthermore, although *srd5a* genes are present in various teleost species (4, 32, 33), details of DHT and 11KDHT production remained unclear in most species. In this study, we compared the profiles, biosynthesis and functions of DHT and 11KDHT in humans and eels, as representatives of mammals and teleosts that possess abundant classical androgens and 11-oxygenated androgens in both sexes (18, 22–24, 34–36).

## Materials and Methods

### Human and Eel Blood Samples

The research protocol using human materials was approved at the ethical committee of Asahikawa Medical University and University of Fukui. Blood samples were collected in collection tubes containing heparin from the median cubital vein of 2 healthy women volunteers (obtaining informed consent) at University of Fukui Hospital in 2007. Plasma was separated by centrifugation at 1000*g* for 5 minutes. Other plasma samples were purchased from AllCells (Alameda, CA, USA) and ProMedDX (Norton, MA, USA). All plasma samples were collected under IRB-approved collection protocols and subject informed consent. The donors were 5 men (aged 32.2 ± 5.5 y) and 5 women (aged 29.2 ± 6.3y).

Animal experiments were performed using protocols approved by the Animal Care and Use Committee of Kumamoto University (Approval Number: A2020-014). All experiments were performed in accordance with the relevant guidelines and regulations. The sexes of juvenile eels were artificially directed by feeding a commercial diet supplemented with or without E2 (37). Male and female eels (>250g body weight) were accumulated for a month to artificial sea water. After accumulation, sexual maturation was induced by the administration of recombinant luteinizing hormone and the crude extracts of commercial salmon pituitary to male and female eels, respectively, as described (38, 39). Blood samples were collected from the animals using a heparinized syringes and needles, and plasma was separated by centrifuging the blood at 1000*g* for 5 minutes.

### Cell Culture, Transfection and Luciferase Assay

HEK293 and CV-1 cells were cultured in DMEM supplemented with 10% fetal bovine serum (FBS) in a humidified atmosphere containing 5% CO_2_/95% air at 37 °C. Hepa-E1 cells were cultured in E-RDF with 5% FBS at 28 °C. Cells were dispensed into 24-well plates at 5 × 10^4^ cells per well 24 h before transfection. HEK293 and CV-1 cells were transfected using HilyMax (Dojindo Laboratories, Kumamoto, Japan) according to the manufacturer’s instructions. Hepa-E1 cells were transfected using Lipofectamine LTX (Thermo Fisher Scientific, Waltham, MA, USA) according to the manufacturer’s instructions. At 24 h or 2 days post-transfection, the cells were treated with vehicle (EtOH) or androgens. Luciferase activity was determined using a dual luciferase reporter assay system (29, 40). Measurements were made using a MiniLumat LB9506 (Berthold Systems, Aliquippa, PA, USA) in a single tube, with the first assay involving the firefly luciferase, followed by the *Renilla* luciferase assay. Firefly luciferase activities (relative light units) were normalized by *Renilla* luciferase activities. Each data point represents the mean of at least three independent experiments.

### Reverse Transcriptase-Polymerase Chain Reaction (RT-PCR) and Quantitative PCR (qPCR)

The cDNA from various human tissues were synthesized as described (26, 41). Total RNA from eel tissues was extracted using TRIsure reagent (Bioline, Luckenwalde, Germany). RT-PCR and qPCR were performed as described (40, 42, 43). The cDNA was synthesized from total RNA of each tissue using SuperScript III Reverse Transcriptase (Thermo Fisher Scientific). The reaction products of the RT-PCR assay were subjected to electrophoresis in a 1.25% agar gel, and the resulting bands were visualized by staining with ethidium bromide. In qPCR, each gene was measured *via* real-time PCR using the LightCycler 480 (Roche Diagnostics, Mannheim, Germany). β-actin (human) and ef1 [eel (44)] were used as the reference genes. Each reaction was conducted in duplicate. As a negative control, template cDNA was replaced by PCR grade water. Relative gene expression levels were determined by using the delta-delta Ct method. The primers used for PCR are described in **Supplementary Table 1** and **Supplementary Figure 2**.

### Cloning of Eel Srd5a cDNAs and Phylogenetic Analysis

Putative nucleotide sequences encoding eel srd5a1, srd5a2a and srd5a2b were provided from the database, JPEEL2016 (http://molas.iis.sinica.edu.tw/jpeel2016/). Cloning of ORF sequences were performed by PCR-based methods using ovary and testis cDNAs.

The alignment analysis of SRD5A/srd5a sequences was performed using Clustal W. The neighbor-joining phylogenetic tree was constructed using MEGA version X. Analyzed proteins and their accession numbers are as follows: human SRD5A1 (NP_001038.1), human SRD5A2 (ABQ59050.1), mouse Srd5a2 (NP_444418.1), chicken Srd5a2 (XP_001235447.1), Japanese quail Srd5a1 (XP_015709859.1), Japanese quail Srd5a2 (XP_015713214.1), three-toed box turtle Srd5a1 (XP_024056462.1), three-toed box turtle Srd5a2 (XP_026505351.1), zebrafish srd5a1 (AAI64429.1), zebrafish srd5a2 (XP_005157051.1), Atlantic salmon srda5a1 (XP_014033435.1), Atlantic salmon srd5a2 (NP_001134686.1), rainbow trout srd5a1 (XP_021428075.1). rainbow trout srd5a2 (XP_021413035.1) and elephant shark srd5a1 (NP_001279361.1).

### Plasmids

The pQCXIP expressing human SRD5A1, human SRD5A2, eel srd5a1, eel srd5a2a and eel srd5a2b were generated by cloning the open reading frame of each gene into a pQCXIP vector (Invitrogen). The pcDNA3 expressing eel arα and arβ were generated by cloning the open reading frame of each gene into a pcDNA vector (Invitrogen, Carlsbad, CA, USA). A Slp-ARU/Luc reporter, pQCXIP/green fluorescent protein (GFP) and pQCXIP/human AR were prepared as described (29).

### Measurements by Liquid Chromatography-Tandem Mass Spectrometry (LC-MS/MS)

Quantification of T, 11-KT, DHT and 11-KDHT in plasma and culture media by LC-MS/MS are based on methods as described [(29), ASKA Pharma Medical Co., Ltd., Kanagawa, Japan]. The lower detection limits of each androgen were 0.25 pg/ml. As internal standards, 11KT-d3, T-13C3, DHT-13C3 and 11KDHT-d3 were added to a medium which was diluted with distilled water. The steroids were extracted with methyl *tert*-butyl ether (MTBE). After the MTBE layer was evaporated to dryness, the extract was dissolved in 0.5 mL of methanol and diluted with 1 ml of distilled water. The sample was applied to OASIS MAX cartridge which had been successively conditioned with 3 ml of methanol and 3 ml of distilled water. After the cartridge was washed with 1 ml of distilled water, 1 ml of methanol/distilled water/acetic acid (45:55:1,v/v/v), and 1 ml of 1% pyridine solution, the steroids were eluted with 1 ml of methanol/pyridine (100:1,v/v). After evaporation, the residue was reacted with 50 μl of mixed solution (80 mg of 2-methyl-6-nitrobenzoic anhydride, 20 mg of 4-dimethylaminopyridine, 40 mg of picolinic acid and 10 μl of triethylamine in 1 ml of acetonitrile) for 30 min at room temperature. After the reaction, the sample was dissolved in 0.5 ml of ethyl acetate/hexane/acetic acid (15:35:1, v/v/v) and the mixture was applied to HyperSep Silica cartridge (Thermo Fisher Scientific) which had been successively conditioned with 3 mL of acetone and 3 ml of hexane. The cartridge was washed with 1 mL of hexane, and 2 mL of ethyl acetate/hexane (3:7, v/v). T, DHT, 11-KDHT and 11-KT were eluted with 2.5 ml of acetone/hexane (7:3, v/v). After evaporation, the residue was dissolved in 0.1 ml of acetonitrile/distilled water (2:3, v/v) and the solution was subjected to a LC-MS/MS.

### Statistical Analysis

Data are presented as the mean ± SEM. Differences between groups (*P*< 0.05) were assessed by the Student’s *t*-test, one-way ANOVA followed by Tukey’s multiple comparison tests and two-way ANOVA followed by Tukey’s multiple comparison tests using SigmaPlot 14 (Systat Software Inc., CA, USA) and EZR (Saitama Medical Center, Jichi Medical University, Saitama, Japan) which is a graphical user interface for R (The R Foundation for Statistical Computing, Vienna, Austria) as described (40).

## Results

### Comparison of DHT and 11-KDHT Levels in Eel and Human Plasma

**Figure 2:**
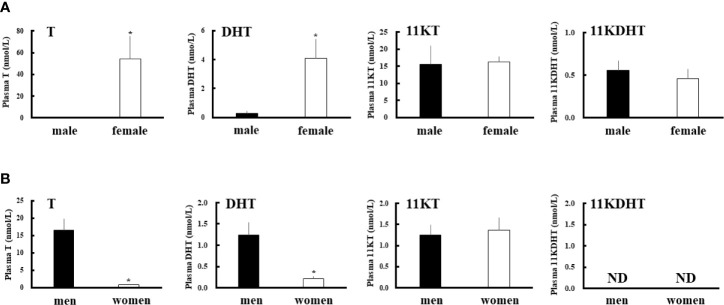
Plasma androgens levels in eels **(A)** and humans **(B)**. Plasma T, DHT, 11-KT and 11-KDHT levels in each sex were measured by LC-MS/MS. Data represent the mean ± SEM (*n* =4 for eels and *n*=5 for humans, for each sex). *P < 0.05 vs male eels or men.

We measured the plasma concentrations of 11-KT, T, DHT and 11-KDHT both in eels and humans (**Figure 2**). In eels, 11-KT levels were similar in the two sexes, whereas the T level was much higher level in females (about 175-fold). Consistent with the results of their precursors, 11KDHT levels were similar between the two sexes, whereas DHT was at much higher concentration in females (about 15-fold). Interestingly, the DHT level in female eels was higher than in human males (**Figure 2B**). As previously reported (45), human plasma T and DHT levels were much higher in males than in females. In humans, plasma 11KT was present in both sexes, whereas 11KDHT was undetectable in both. These observations suggest that although DHT is an androgen common to both eels and humans, 11KDHT is a teleost-specific androgen. Furthermore, the sexual dimorphism of plasma DHT and T levels is reversed between humans and eels.

### Comparison of Expression and Enzymatic Activities of Eel Srd5a and Human SRD5A Genes

**Figure 3:**
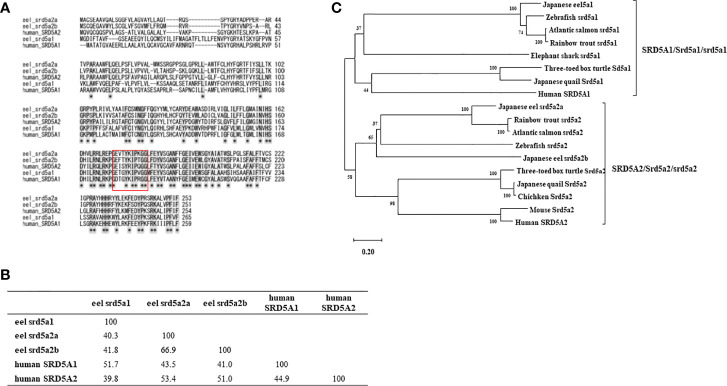
Alignment **(A)** and identities of amino acid sequence **(B)**, phylogenic analyses **(C)** for eel srd5a and human SRD5A. **(A)** Alignment of the deduced SRD5A/srd5a amino acid sequences of eel and human. Conserved NADPH-binding domain (GXXGXXXXXGG) is shown by a box. **(B)** Comparisons of deduced amino acid identities between eel srd5a1, eel srd5a2a, eel srd5a2b, human SRD5A1 and human SRD5A2. **(C)** The phylogenetic tree of SRD5A/Srd5a/srd5a proteins. Bootstrap values (100 resamplings) are indicated by numbers.

To reveal DHT and 11KDHT biosynthesis pathways from T and 11KT in teleost, respectively, eel srd5a genes were cloned from testis and ovary cDNA templates by RT-PCR. A single isoform *srd5a1* and two isoforms of *srd5a2* (*srd5a2a* and *srd5a2b*) genes were isolated in eel (**Supplementary Figure 1**, **Figure 3A**). Eel srd5a1 cDNA (DDBJ accession number: LC602244) comprised an open reading frame (ORF) of 798 bp encoding 265 amino acids (aa) (**Supplementary Figure 1A**, **Figure 3A**). It shared approximately 50% and 40% aa identities with human SRD5A1 and SRD5A2/Srd5a2, respectively (**Figure 3B**). Eel srd5a2a (DDBJ accession number: LC602245) and srd5a2b (DDBJ accession number: LC602246) cDNAs comprised ORFs of 762 and 756 bp encoding 253 and 251 aa, respectively (**Supplementary Figures 1B, C**, **Figure 3A**). These isoforms shared comparatively high similarities (66.9%) with each other. On the other hand, srd5a2a and srd5a2b shared 50% and 40% aa identities with human SRD5A2 and SRD5A1/srd5a1, respectively. Alignments of these proteins revealed the conservation of an aa sequence with the C-terminus (**Figure 3A**). Phylogenetic analysis showed that each of the srd5a genes was included in the corresponding cluster of vertebrate SRD5A/Srd5a/srd5a genes (**Figure 3C**). The analysis also suggested that among the two srd5a2 isoforms, srd5a2b probably represents an ancestral form of vertebrate SRD5A2/Srd5a2/srd5a2.

**Figure 4:**
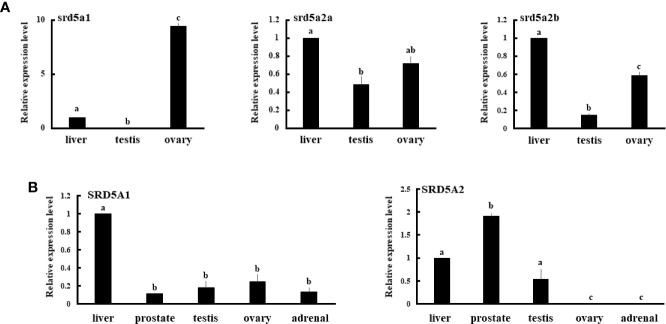
Expression of eel srd5a genes **(A)** and human SRD5A genes **(B)** in various tissues. mRNA expression of each gene in each tissue was analyzed by qPCR and normalized to ef-1 **(A)** or β-actin **(B)** expression. Data represent the mean ± SEM of at least three independent samples. Values marked by different letters are significantly different (*P*< 0.05).

Tissue expression analyses of mRNA by qPCR revealed that eel srd5a1 was expressed in liver and ovary, but not in testis (**Figure 4A**). Both srd5a2 isoforms were expressed in liver, testis and ovary, although ovarian srd5a2b expression levels were significantly higher than testicular levels. Human SRD5A1 was expressed in all examined tissues including the primary steroidogenic tissues, testis, ovary and adrenal (**Figure 4B**). In contrast SRD5A2 was expressed only in liver, prostate and testis (**Figure 4B**).

**Figure 5:**
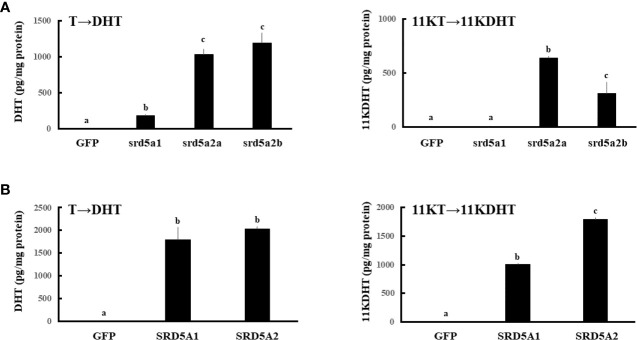
Evaluation of the enzymatic activities of eel srd5a **(A)** and human SRD5A **(B)**. Expression vectors of each gene were transfected, and 24h after transfection, cells were incubated with T (1 nM) or 11KT (1 nM) for 3h. Concentrations of DHT and 11KDHT in culture media were analyzed by LC-MS/MS. Each column represents the mean ± SEM (*n* =3 for each group) of three independent experiments. Values marked by different letters are significantly different (*P*< 0.05).

To investigate the enzymatic activities of SRD5A/srd5a isoforms for conversion of T and 11KT to DHT and 11KDHT, respectively, expression vectors of GFP and each of SRD5A/srd5a genes were transfected in HEK293 cells. Then, T or 11KT was added to the culture medium at 10^-9^ M for 3h. All eel srd5a and human SRD5A isoforms catalyzed the conversion of T into DHT (**Figure 5A**). In the eel, those activities were significantly higher in both srd5a2 isoforms than in srd5a1. Conversion of 11KT into 11KDHT was catalyzed by both eel srd5a2 isoforms and human SRD5A (**Figure 5B**). Eel srd5a2a and human SRD5A2 had significant higher activities than eel srd5a2b and human SRD5A1, respectively. In contrast, eel srd5a1 showed no activity for conversion 11KT to 11KDHT.

### Effects of DHT and 11-KDHT on AR/ar-Mediated Transactivation

**Figure 6:**
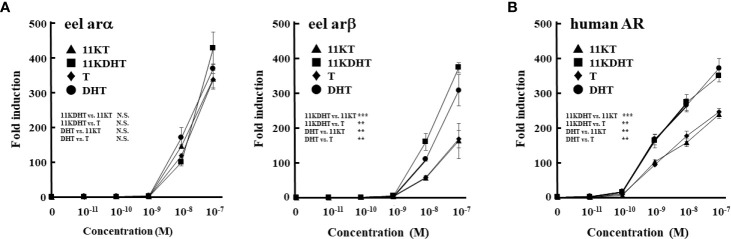
Eel ars **(A)** and human AR **(B)**-mediated transactivation by each androgen in Hepa-E1 cells and CV-1 cells, respectively. Hepa-E1 cells and CV-1 cells were transfected with the ARE-Luc vector and each ar/AR-expression vector. At 24 h post-transfection, cells were incubated with or without increasing concentrations of 11KT, 11KDHT, T and DHT for 24 h. Data represent the mean ± sem of at least three independent experiments. Results of two-way ANOVA (the additives and their concentrations as factors) followed by multiple comparison tests are given (N.S. *P*≧0.05; **P < 0.01; ***P < 0.001).

To investigate the effects of 5α-reduced androgens on eel ars (arα and arβ) and mammalian AR, we compared the androgen-dependent transcriptional activities of DHT and 11KDHT with the activities of T and 11KT using the luciferase reporter system in fish and mammalian cell lines (**Figure 6**). DHT and 11KDHT at >10^-8^ M activated eel arα-mediated transactivation in a similar manner to that of T and 11KT (**Figure 6A**). DHT and 11KDHT at >10^-8^ M also activated eel arβ, although to a greater extent than T and 11KT (**Figure 6A**). Human AR-mediated transactivation was increased by DHT and 11KDHT above 10^-10^ M in a concentration-dependent manner (**Figure 6B**). Both were stronger activators of human AR than T and 11KT, paralleling the response of eel arβ. These results indicate that 5α-reduced androgens strongly activate AR/ars-mediated transactivation in both humans and teleosts.

## Discussion

This study demonstrated that DHT and 11KDHT are potential important androgens in eels. In contrast, 11KDHT was undetectable in healthy human plasma, although DHT was present.

DHT plays very important roles in mammalian sex differentiation as the most potent androgen (4). Mutations of the *SRD5A2* gene cause 46, XY disorders of sex development, resulting from low DHT production (4, 8). In contrast, little attention has been received in the existence and functions of DHT in fish, because it is thought to be relatively biologically inactive. Therefore, it is unexpectable that plasma DHT levels in eels were higher than those in men. In contrast to humans and other mammals, it was the female-dominated androgen in eels. These differences probably reflect the opposite sexual dimorphism of T levels, as a precursor to DHT. This result is consistent with a previous report that plasma T concentrations of wild females become higher than those of males during silvering, prepubertal stage in eels (46). T is the predominant male androgen in mammals, whereas plasma profiles of T are species-specific among teleosts (35); T is often the predominant androgen both males and females. Therefore, it is possible that the profiles of plasma DHT differ among teleost species. Until now, male-dominant androgens are never reported at least in sexually matured eels. T and DHT are necessary for regulating various male physiological functions in humans during long reproductive age ensuing puberty (1). In contrast, male eels are considered to die after their first spawning following the sexual maturation (47). Therefore, androgens like T and DHT in humans might be unnecessary in maturated male eels. This hypothesis might be supported by the facts that plasma T levels in females are abundant in the salmons [also die after their first spawning (48)]. In contrast to DHT, 11KDHT levels did not show any sexual differences. This might also result from the absence of sexual dimorphism of precursor (11KT) levels. 11-oxygenated androgens levels including 11KT are very low in most female teleosts, except in eels, sturgeon, salmonids and mullet (35). Therefore, 11KDHT could be the dominant male androgen in most teleosts. Regardless of the differences in sexual plasma profiles, DHT and 11KDHT strongly activate various teleost ars [**Figure 6** (49, 50)], which suggest that DHT and 11KDHT are important androgens in teleost species. Future study should investigate the plasma profiles of these 5α-reduced androgens in many teleost species. In contrast to eels, 11KDHT is not present in healthy human plasma, even though its precursor 11KT exists. This is likely owing to the deficiency of SRD5A2 expression (main converting enzyme from 11KT to 11KDHT) in the adrenal glands, which are the main source of 11-oxygenated androgens in humans (36). However, 11KDHT is detectable in prostate tissues and plasma samples of prostate cancer patients (31). Thus, the possibility that 11KDHT functions in humans under the pathological conditions should not be ruled out.

In addition to a single SRD5A1 homolog, there are two SRD5A2 homologs in eels, which showed relatively similar activities for production of 5α-reduced androgens. These srd5a2 paralogs perhaps occurred by the teleost-specific genome duplication. Although such srd5a2 paralogs in some species are registered in databases, their prevalence and general importance among Teleostei are unclear. In the future study, it is necessary to investigate the presence of srd5a2 paralogs in teleosts. Consistent with our results, previous studies reported that human SRD5A2 showed higher activities than SRD5A1 in converting T and 11KT into DHT and 11KDHT, respectively (9, 30). Such difference between SRD5A/Srd5a/srd5a isoforms is probably conserved during evolution, despite eel srd5a1 being completely inactive in converting 11KT to 11KDHT. In addition to studying the profiles of 5α-reduced androgens, it would be interesting to evaluate the activities of SRD5A/Srd5a/srd5a homologs in various animal species to reveal the transitions of these androgens during evolution.

The 5α-reduced androgens, DHT and 11KDHT more strongly activated human AR and eel arβ than T and 11KT did, whereas such remarkable differences were not detected for eel arα. In teleosts, the two ar genes have been formed by duplication of an ancestral AR gene during a teleost specific genome duplication event. Based on the phylogenic analyses, Ogino and colleagues proposed that teleost arβ preserves the ancestral AR functions, whereas arα has acquired new properties during more rapid evolution (28). Our results are consistent with this hypothesis; i.e. eel arα has lost its preference for the 5α-reduced androgens. In human, DHT is essential for various physiological phenomena as a stronger activator of AR than T, despite plasma concentrations of DHT are much lower than those of T (4). Therefore, it is possible that in eel, DHT and 11KDHT are especially important in arβ-expressing tissues, including testis and ovary (47, 49, 51). In contrast, previous studies have suggested that 11KT is the predominant androgen activating teleost ars (28, 52, 53). Although DHT activates almost teleost ars, the activation is often reported to be weaker than with 11KT, including the case of eel arβ (49). Such discrepancy reflects the different cell lines used in those studies. Previous studies have used mammalian cell lines. In the preset study, we used Japanese eel hepatocyte-derived Hepa-E1 cells for measuring the eel ar-mediated transactivation. In support of the above hypothesis, activation of eel ars by each of the androgens was very low (less than 10-fold relative to vehicle groups) in CV-1 cells even at 10^-7^ M (data not shown), whereas it was over several hundred-fold higher in Hepa-E1 cells, at levels similar to that of human AR in CV-1 cells. A similar phenomenon was noted in measurements of flounder estrogen receptor-mediated transactivation by estrogens and estrogenic compounds (54). Because most teleost transcription factors are strongly activated in reporter assays using mammalian cells, sex steroid receptors might be an exception in poorly activated in mammalian cells. Future studies should compare the effects of T, 11KT, DHT and 11KDHT on teleost ar-mediated transactivation using fish cell lines.

In summary, we demonstrated that DHT and 11KDHT are potent endogenous androgens in fish. The properties of these androgens could provide useful insights into elucidating ambiguous ar-mediated phenomena reported in fish. In contrast, 11KDHT is undetectable in healthy humans. Nevertheless, it has been often reported that the profiles of 11-oxygenated androgens are markedly changed under pathological conditions (36, 55–59). It is important to investigate the presence of 11KDHT in these androgen-dependent diseases to seek novel targets for therapy.

## Data Availability Statement

‘The raw data supporting the conclusions of this article will be made available by the authors, without undue reservation.

## Ethics Statement

The studies involving human participants were reviewed and approved by The ethical committee of Asahikawa Medical University and University of Fukui. The patients/participants provided their written informed consent to participate in this study. The animal study was reviewed and approved by The Animal Care and Use Committee of Kumamoto University.

## Author Contributions

TY, YI, TSe, JU, ST, NS, AU and TK obtained funding and designed the study. TY, HI, YI, TSe, JU, MI, MO, DM, TI, TSa, YM and TK performed the experiments and collected the data. TY, HI, YI, MK and TK wrote the manuscript. All authors contributed to the article and approved the submitted version.

## Conflict of Interest

Author YM was employed by the company ASKA Pharmaceutical Medical Co., Ltd.

The remaining authors declare that the research was conducted in the absence of any commercial or financial relationships that could be construed as a potential conflict of interest.
