# Supplementary material for: Profiles of 5α-Reduced Androgens in Humans and Eels: 5α-Dihydrotestosterone and 11-Ketodihydrotestosterone Are Active Androgens Produced in Eel Gonads
Source: Front Endocrinol (Lausanne). 2021 Mar 23;12:657360. doi: 10.3389/fendo.2021.657360 (PMC8021924; doi:10.3389/fendo.2021.657360)
Supplement: Supplementary file 1 [file DataSheet_1.pdf]

Supplementary Table 1. Primers used in each experiment.

PCR primers	Forward primers	Reverse primers
**qPCR (efficiency %)**		
human β-actin (98.9)	F- ggacttcgagcaagagatgg	R- aaggaaggctggaagagtgc
human SRD5A1 (96.7)	F- tggcgattatgttctgtacctgta	R- aaccacaagccaaaacctattaga
human SRD5A2 (96.6)	F- cggtttagcttgggtgtcttcttatt	R- tggctccagaaacatacgtaaacaag
eel ef1 (95.8)	F- tgtgggagtcaacaagatgga	R- ctcaaaacgcttctggctgta
eel srd5a1 (95.2)	F- gttcggttcagaagctgagg	R- ccataggggacgttctcaaa
eel srd5a2a (92.8)	F- gcagccatattctgctccat	R- ccattcccaggaagaacaga
eel srd5a2b (94.5)	F- ggcttcatccagggtcacta	R- tttctcaggtttcgcaggat
