# Supplementary material for: Profiles of 5α-Reduced Androgens in Humans and Eels: 5α-Dihydrotestosterone and 11-Ketodihydrotestosterone Are Active Androgens Produced in Eel Gonads
Source: Front Endocrinol (Lausanne). 2021 Mar 23;12:657360. doi: 10.3389/fendo.2021.657360 (PMC8021924; doi:10.3389/fendo.2021.657360)
Supplement: Supplementary file 2 [file DataSheet_2.pdf]

**A** **srd5a1**

```
  1 ATGGGGGATATCTTTACCGCGGTGTTCGGTTCAGAAGCTGAGGAG
     M  G  D  I  F  T  A  V  F  G  S  E  A  E  E
 46 CAATATATACTACAATGCATGTCTTACATTCTAATATTCATGGCC
     Q  Y  I  L  Q  C  M  S  Y  I  L  I  F  M  A
 91 GGGGCAACATTTTTAACTTTACTTTTTGAGAACGTCCCCTATGGC
     G  A  T  F  L  T  L  L  F  E  N  V  P  Y  G
136 AGATATGCAACCAGCAAATATGGGTTTCCTGTCAACGTTAAACTT
     R  Y  A  T  S  K  Y  G  F  P  V  N  V  K  L
181 GCATGGTTTGTGCAGGAGTTGCCTGCATTCGTGTTACCCGTCTTT
     A  W  F  V  Q  E  L  P  A  F  V  L  P  V  F
226 TTAGTGCTGAAGTCGTCTGCTGCACAGTTATCTGAGACGGCAAAT
     L  V  L  K  S  S  A  A  Q  L  S  E  T  A  N
271 CGTTTTCTTATCGCTATGTATTTTTGTCATTATGTGCACAGATCT
     R  F  L  I  A  M  Y  F  C  H  Y  V  H  R  S
316 CTCGTCTACCCGTTTTTGATTAGAGGAGGAAAACCAACCCCCTTT
     L  V  Y  P  F  L  I  R  G  G  K  P  T  P  F
361 TTTTCGTTTGCCCTGGCGTTTGTTTTCTGTATATACAATGGGTAT
     F  S  F  A  L  A  F  V  F  C  I  Y  N  G  Y
406 CTCCAGATACGACACCTGAGCCACTTTGCAGAGTACCCTAAAGAC
     L  Q  I  R  H  L  S  H  F  A  E  Y  P  K  D
451 TGGGTCAGACACCCTTGGTTCATCGCCGGATTCGTGCTCTGGTTA
     W  V  R  H  P  W  F  I  A  G  F  V  L  W  L
496 TTAGGGTGGCTTGTAAACGTCCATTCAGACCATATCCTTCGAAAC
     L  G  W  L  V  N  V  H  S  D  H  I  L  R  N
541 CTTCGAAAACCGGGGGAAACAGGTTATAAGATACCAGTAGGTGGC
     L  R  K  P  G  E  T  G  Y  K  I  P  V  G  G
586 ATGTTTGAATATGTGTCAGGCGCAAATTTCCTGGGGGAAATTGTG
     M  F  E  Y  V  S  G  A  N  F  L  G  E  I  V
631 GAGTGGTCAGGATTTGCTCTTGCGGCTCACTCAATTCACAGTGCT
     E  W  S  G  F  A  L  A  A  H  S  I  H  S  A
676 GCTTTTGCCATCTTTACGTTCGTCGTGCTTTCCAGCAGGGCTGTG
     A  F  A  I  F  T  F  V  V  L  S  S  R  A  V
721 GCTCATCACAAGTGGTACCTTGCAAAATTTGAAGACTACCCAAAG
     A  H  H  K  W  Y  L  A  K  F  E  D  Y  P  K
766 TCAAGAAAAGCATTAATACCTTTTGTATTTTGA
     S  R  K  A  L  I  P  F  V  F  *
```

**B** **srd5a2a**

```
  1 ATGGCGTGTAGCGAGGCGGCGGTGCAGGCCCTCAGCGGCGGCTTC
     M  A  C  S  E  A  A  V  Q  A  L  S  G  G  F
 46 GTCCTGGCGGGCGTGGCGTACCTCCTGGCACAGACGCGGCAGTCC
     V  L  A  G  V  A  Y  L  L  A  Q  T  R  Q  S
 91 AGCCCCTACGGGCGCTACGCGGACCCCCCCGAGCGCGCCCGCACC
     S  P  Y  G  R  Y  A  D  P  P  E  R  A  R  T
136 GTGCCCGCCCGGGCGGCCTGGTTCCTCCAGGAGCTCCCCTCTTTC
     V  P  A  R  A  A  W  F  L  Q  E  L  P  S  F
181 CTGGTCCCCGTGGCCCTGATGTGGAGCTCGCGCGGACCCCCCTCC
     L  V  P  V  A  L  M  W  S  S  R  G  P  P  S
226 GGGCTGGGACCCCGGCTGCTCGCCTGGACCTTCTGCCTCCATTAC
     G  L  G  P  R  L  L  A  W  T  F  C  L  H  Y
271 TTCCAGAGGACATTCATCTATTCCTTGCTGACCAAAGGTCGTCCG
     F  Q  R  T  F  I  Y  S  L  L  T  K  G  R  P
316 TACCCTCTGCGCATAGTCCTTTACGCAGCCATATTCTGCTCCATG
     Y  P  L  R  I  V  L  Y  A  A  I  F  C  S  M
361 AACGGCTTCTTCCAAGGCTACTACATGCTATACTGCGCCCGGTAT
     N  G  F  F  Q  G  Y  Y  M  L  Y  C  A  R  Y
406 GACGAGGCCTGGGCGTCCGACATCCGCCTGGTTATCGGACTGATT
     D  E  A  W  A  S  D  I  R  L  V  I  G  L  I
451 CTGTTCTTCCTGGGAATGGCCATCAATATCCACAGCGATCATGTT
     L  F  F  L  G  M  A  I  N  I  H  S  D  H  V
496 CTGCGCCGTTTGAGAGAACCCGGTGAAGTCACTTACAAGATTCCC
     L  R  R  L  R  E  P  G  E  V  T  Y  K  I  P
541 AAAGGGGGCTTGTTTGAGTACGTCTCGGGTGCCAACTTCTTTGGG
     K  G  G  L  F  E  Y  V  S  G  A  N  F  F  G
586 GAGATAGTGGAATGGTCCGGCTACGCCATAGCAACCTGGTCGCTG
     E  I  V  E  W  S  G  Y  A  I  A  T  W  S  L
631 CCAGGCCTCTCCTTCGCCCTCTTCACCGTGTGCTCCATTGGACCG
     P  G  L  S  F  A  L  F  T  V  C  S  I  G  P
676 CGCGCCTACCATCATCACAGGTATTACCTGGAAAAATTTGAGGAT
     R  A  Y  H  H  H  R  Y  Y  L  E  K  F  E  D
721 TACCCCAGGTCCAGGAAGGCTTTGGTGCCGTTCATCTTCTGA
     Y  P  R  S  R  K  A  L  V  P  F  I  F  *
```

**C** **srd5a2b**

```
  1 ATGTCGTGCCAGGAAGGCGCCGTGTGGTACCTCAGCTGCGGGCTG
     M  S  C  Q  E  G  A  V  W  Y  L  S  C  G  L
 46 GTCTTCAGCGGGGTGATGTTCCTCTTCAGACAGATGAGGGTCCGC
     V  F  S  G  V  M  F  L  F  R  Q  M  R  V  R
 91 ACTCCCTACGGGCGCTACGTGAACCCCTCTGCGAGGTTGCTGGTG
     T  P  Y  G  R  Y  V  N  P  S  A  R  L  L  V
136 CCGGCCAAGTTGGCTTGGTTCCTCCAGGAGCTGCCCTCCTTGCTG
     P  A  K  L  A  W  F  L  Q  E  L  P  S  L  L
181 GTCCCTGTGGTTCTGGTCCTGACGGCACACTCTCCATCCAAACTG
     V  P  V  V  L  V  L  T  A  H  S  P  S  K  L
226 GGACAGAAGCTTCTGCTCTGGACGTTCTGCCTGCACTACTTCCAG
     G  Q  K  L  L  L  W  T  F  C  L  H  Y  F  Q
271 AGGACATTCATCTTTTCGTTGCTGACCAAAGGCCGCCCTTCCCCT
     R  T  F  I  F  S  L  L  T  K  G  R  P  S  P
316 CTCAAGATTGTAGTCTCCGCTACTGCGTTCTGCTCCATCAACGGC
     L  K  I  V  V  S  A  T  A  F  C  S  I  N  G
361 TTCATCCAGGGTCACTATCACCTCCACTGCTTCCAGTATACGGAG
     F  I  Q  G  H  Y  H  L  H  C  F  Q  Y  T  E
406 GTCTGGCTGGCGGACATCTGCCTTGTGGCTGGTTTGATATTATTC
     V  W  L  A  D  I  C  L  V  A  G  L  I  L  F
451 TTTCTGGGAATGGCCATCAACGTCCACAGCGACCACATCCTGCGA
     F  L  G  M  A  I  N  V  H  S  D  H  I  L  R
496 AACCTGAGAAAACCAGGCGAATTCACCTACAAAATTCCAACAGGT
     N  L  R  K  P  G  E  F  T  Y  K  I  P  T  G
541 GGTCTGTTTGATTACGTCTCCGGTGCCAACTTCCTCGGTGAGATC
     G  L  F  D  Y  V  S  G  A  N  F  L  G  E  I
586 GTGGAATGGTTGGGCTACGCGGTCGCTACTCGGTCCTTTCCCGCG
     V  E  W  L  G  Y  A  V  A  T  R  S  F  P  A
631 ATCTCCTTCGCCTTTTTTACCATGTGCTCCATAGGACCGCGAGCC
     I  S  F  A  F  F  T  M  C  S  I  G  P  R  A
676 TACCACCACCACAGGTTCTACAAAGAGAAATTCAGCGATTACCCA
     Y  H  H  H  R  F  Y  K  E  K  F  S  D  Y  P
721 CAGTCCAGGAAAGCTTTGGTCCCTTTCATCTTTTGA
     Q  S  R  K  A  L  V  P  F  I  F  *
```

**Supplementary Fig. 1** . Nucleotide and deduced amino acid sequences of Japanese eel srd5a1(A), srd5a2a (B) and srd5a2b (C).

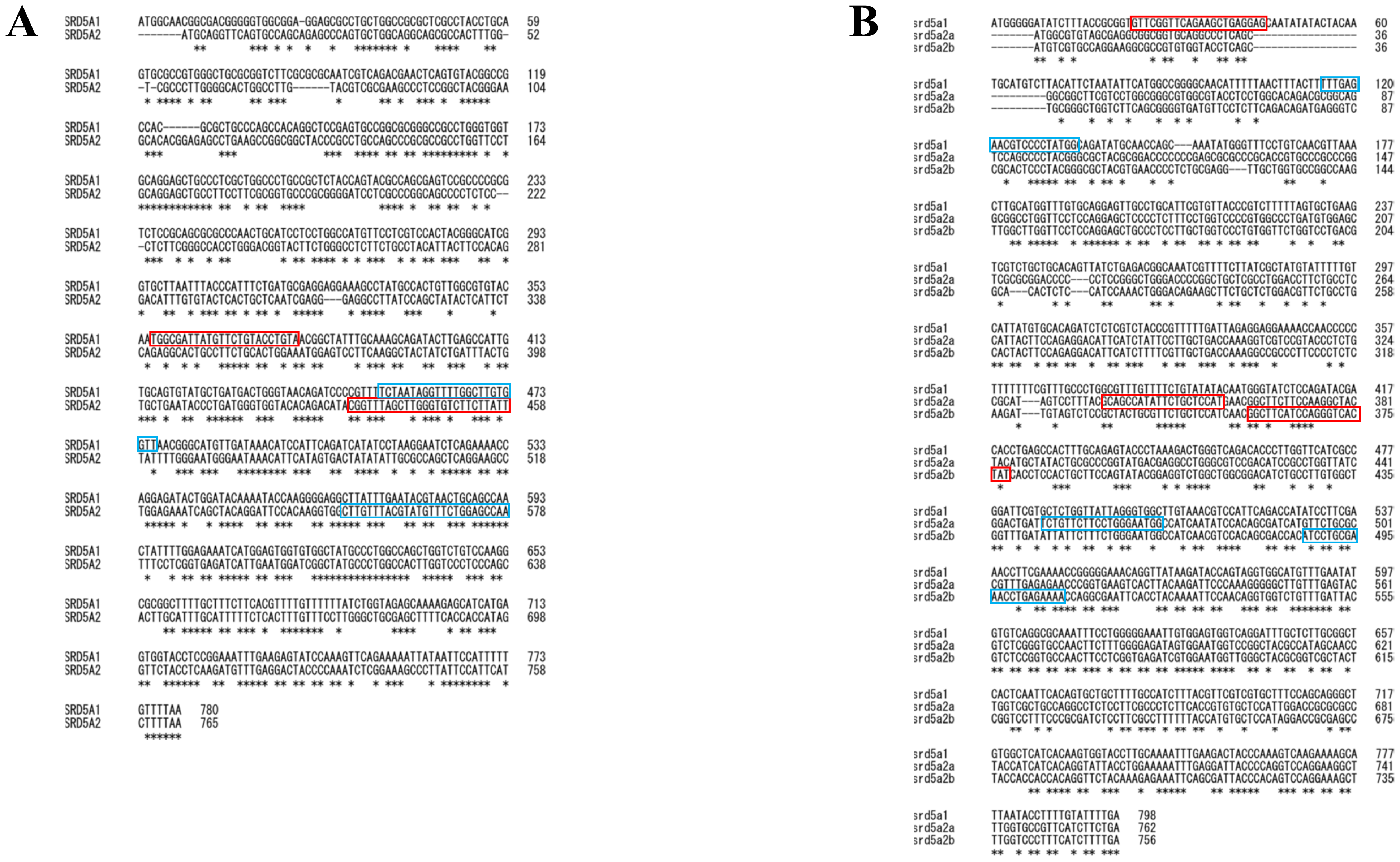


**Supplementary Fig. 2** Alignment of the nucleotide sequences of human SRD5A (A) and eel srd5a (B). Primers used in qPCR analyses are shown by the red (forward) and blue (reverse) boxes.
